# Assembling a global database of child pneumonia studies to inform WHO pneumonia management algorithm: Methodology and applications

**DOI:** 10.7189/jogh.12.04075

**Published:** 2022-12-29

**Authors:** Helena Martin, Jennifer Falconer, Emmanuel Addo-Yobo, Satinder Aneja, Luis Martinez Arroyo, Rai Asghar, Shally Awasthi, Salem Banajeh, Abdul Bari, Sudha Basnet, Ashish Bavdekar, Nita Bhandari, Shinjini Bhatnagar, Zulfiqar A Bhutta, Abdullah Brooks, Mandeep Chadha, Noel Chisaka, Monidarin Chou, Alexey W Clara, Tim Colbourn, Clare Cutland, Valérie D'Acremont, Marcela Echavarria, Angela Gentile, Brad Gessner, Christopher J. Gregory, Tabish Hazir, Patricia L. Hibberd, Siddhivinayak Hirve, Shubhada Hooli, Imran Iqbal, Prakash Jeena, Cissy B Kartasasmita, Carina King, Romina Libster, Rakesh Lodha, Juan M Lozano, Marilla Lucero, Norman Lufesi, William B MacLeod, Shabir Ahmed Madhi, Joseph L Mathew, Irene Maulen-Radovan, Eric D McCollum, Greta Mino, Charles Mwansambo, Mark I Neuman, Ngoc Tuong Vy Nguyen, Marta C Nunes, Pagbajabyn Nymadawa, Kerry-Ann F O'Grady, Jean-William Pape, Glaucia Paranhos-Baccala, Archana Patel, Valentina Sanchez Picot, Mala Rakoto-Andrianarivelo, Zeba Rasmussen, Vanessa Rouzier, Graciela Russomando, Raul O Ruvinsky, Salim Sadruddin, Samir K. Saha, Mathuram Santosham, Sunit Singhi, Sajid Soofi, Tor A Strand, Mariam Sylla, Somsak Thamthitiwat, Donald M Thea, Claudia Turner, Philippe Vanhems, Nitya Wadhwa, Jianwei Wang, Syed MA Zaman, Harry Campbell, Harish Nair, Shamim Ahmad Qazi, Yasir Bin Nisar, World Health Organization Pneumonia Research Partnership to Assess WHO Recommendation (PREPARE) study group

**Affiliations:** 1 Centre for Global Health, Usher Institute, Edinburgh Medical School, University of Edinburgh, Edinburgh, United Kingdom; 2 Kwame Nkrumah University of Science and Technology/Komfo Anokye Teaching Hospital, Kumasi, Ghana; 3 School of Medical Sciences and Research, Sharda University, Greater Noida, India; 4 PNUD/National University, Montevideo, Uruguay; 5 Rawalpindi Medical College, Rawalpindi, Pakistan; 6 King George’s Medical University, Department of Pediatrics, Lucknow, India; 7 Department of Paediatrics and Child Health, University of Sana’a, Sana’a, Yemen; 8 Independent newborn and child health consultant, Islamabad, Pakistan; 9 Center for Intervention Science in Maternal and Child Health, University of Bergen, Norway; 10 Department of Pediatrics, Tribhuvan University Institute of Medicine, Nepal; 11 King Edward Memorial (KEM) Hospital Pune, Department of Pediatrics, Pune, India; 12 Center for Health Research and Development, Society for Applied Studies, India; 13 Translational Health Science and Technology Institute, Faridabad, India; 14 Institute for Global Health and Development, Aga Khan University, Pakistan,; 15 Department of International Health, Johns Hopkins Bloomberg School of Public Health, Baltimore, Maryland, USA; 16 Former Scientist, Indian Council of Medical Research (ICMR), National Institute of Virology, Pune, India; 17 World Bank, Washington DC, USA; 18 University of Health Sciences, Rodolphe Mérieux Laboratory, Phom Phen, Cambodia; 19 Ministry of Environment, Phom Phen, Cambodia; 20 Centers for Disease Control, Central American Region, Guatemala City, Guatemala; 21 Institute for Global Health, University College London, London, United Kingdom; 22 South African Medical Research Council, Vaccines and Infectious Diseases Analytics Research Unit, School of Pathology, Faculty of Health Sciences, University of the Witwatersrand, Johannesburg, South Africa; 23 Department of Science and Technology/National Research Foundation, South African Research Chair Initiative in Vaccine Preventable Diseases, Faculty of Health Sciences, University of the Witwatersrand, Johannesburg, South Africa; 24 Swiss Tropical and Public Health Institute, Basel, Switzerland; 25 Clinical Virology Unit, Centro de Educación Médica e Investigaciones Clínicas, Argentina; 26 Department of Epidemiology, “R. Gutiérrez” Children's Hospital, Buenos Aires, Argentina; 27 Pfizer Vaccines, Collegeville, Pennsylvania, USA; 28 Division of Vector-borne Diseases, US Centers for Disease Control and Prevention, Fort Collins, Colorado, USA; 29 Retired from Children Hospital, Pakistan Institute of Medical Sciences, Islamabad, Pakistan; 30 Department of Global Health, Boston University School of Public Health, Boston, Massachusetts, USA; 31 King Edward Memorial (KEM) Hospital Research Center, Pune, India; 32 Section of Pediatric Emergency Medicine, Texas Children’s Hospital, Baylor College of Medicine, Houston, Texas, USA; 33 Department of Paediatrics, Combined Military Hospital Institute of Medical Sciences, Multan, Pakistan; 34 University of KwaZulu-Natal, Durban, South Africa; 35 Department of Child Health, Faculty of Medicine, Universitas Padjadjaran, Bandung, Indonesia; 36 Department of Global Public Health, Karolinska Institutet, Stockholm, Sweden; 37 Institute for Global Health, University College London, London, United Kingdom; 38 Fundacion INFANT, Buenos Aires, Argentina; 39 All India Institute of Medical Sciences, New Delhi, India; 40 Florida International University, Miami, USA; 41 Research Institute for Tropical Medicine, Manila, Philippines; 42 Ministry of Health, Lilongwe, Malawi; 43 Faculty of Health Sciences, University of the Witwatersrand, Johannesburg; 44 Advanced Pediatrics Centre, Postgraduate Institute of Medical Education and Research, Chandigarh, India; 45 Instituto Nactional de Pediatria Division de Investigacion Insurgentes, Mexico City, Mexico; 46 Global Program in Respiratory Sciences, Eudowood Division of Pediatric Respiratory Sciences, Department of Pediatrics, Johns Hopkins School of Medicine, Baltimore, USA; 47 Department of Infectious diseases, Guayaquil, Ecuador; 48 Division of Emergency Medicine, Boston Children’s Hospital, Harvard Medical School, Boston, Massachusetts, USA; 49 Children Hospital No 1, Ho Chi Minh City, Vietnam; 50 Mongolian Academy of Sciences, Academy of Medical Sciences, Ulaanbaatar, Mongolia; 51 Australian Centre for Health Services Innovation, Queensland University of Technology, Kelvin Grove, Australia; 52 GHESKIO Center, Port au Prince, Haiti; 53 Fondation Merieux, Lyon, France; 54 Lata Medical Research Foundation, Nagpur and Datta Meghe Institute of Medical Sciences, Sawangi, India; 55 Centre d'Infectiologie Charles Mérieux, Antananarivo, Madagascar; 56 Division of International Epidemiology and Population Studies (DIEPS), Fogarty International Center (FIC), National Institute of Health (NIH), USA; 57 Universidad Nacional de Asuncion, Departamento de Biología Molecular y Genética, Instituto de Investigaciones en Ciencias de la Salud, Asuncion, Paraguay; 58 Dirección de Control de Enfermedades Inmunoprevenibles, Ministerio de Salud de la Nación, Buenos Aires, Argentina; 59 Consultant/Retired World Health Organization (WHO) Staff, Geneva, Switzerland; 60 Child Health Research Foundation, Dhaka, Bangladesh; 61 Dhaka Shishu Hospital, Dhaka, Bangladesh; 62 International Vaccine Access Center (IVAC), Department of International Health, Johns Hopkins University, Baltimore, Maryland, USA; 63 Medanta, The Medicity, Gurgaon, India; 64 Department of Pediatrics and Child Health, Aga Khan University, Pakistan; 65 Research Department, Innlandet Hospital Trust, Lillehammer, Norway; 66 Gabriel Touré Hospital, Department of Pediatrics, Bamako, Mali; 67 Division of Global Health Protection, Thailand Ministry of Public Health – US Centers for Disease Control and Prevention Collaboration, Nonthaburi, Thailand; 68 Shoklo Malaria Research Unit, Mae Sot, Thailand; 69 Unité d'Hygiène, Epidémiologie, Infectiovigilance et Prévention, Hospices Civils de Lyon, Lyon, France; 70 Centre International de Recherche en Infectiologie, École Nationale Supérieure de Lyon, Université Claude Bernard Lyon 1, Lyon, France; 71 Chinese Academy of Medical Sciences & Peking Union, Medical College Institute of Pathogen Biology, MOH Key Laboratory of Systems Biology of Pathogens and Dr Christophe Mérieux Laboratory, Beijing, China; 72 Liverpool School of Tropical Medicine, Liverpool, United Kingdom; 73 Department of Maternal, Newborn, Child and Adolescent Health and Ageing, World Health Organization (WHO), Geneva, Switzerland

## Abstract

**Background:**

The existing World Health Organization (WHO) pneumonia case management guidelines rely on clinical symptoms and signs for identifying, classifying, and treating pneumonia in children up to 5 years old. We aimed to collate an individual patient-level data set from large, high-quality pre-existing studies on pneumonia in children to identify a set of signs and symptoms with greater validity in the diagnosis, prognosis, and possible treatment of childhood pneumonia for the improvement of current pneumonia case management guidelines.

**Methods:**

Using data from a published systematic review and expert knowledge, we identified studies meeting our eligibility criteria and invited investigators to share individual-level patient data. We collected data on demographic information, general medical history, and current illness episode, including history, clinical presentation, chest radiograph findings when available, treatment, and outcome. Data were gathered separately from hospital-based and community-based cases. We performed a narrative synthesis to describe the final data set.

**Results:**

Forty-one separate data sets were included in the Pneumonia Research Partnership to Assess WHO Recommendations (PREPARE) database, 26 of which were hospital-based and 15 were community-based. The PREPARE database includes 285 839 children with pneumonia (244 323 in the hospital and 41 516 in the community), with detailed descriptions of clinical presentation, clinical progression, and outcome. Of 9185 pneumonia-related deaths, 6836 (74%) occurred in children <1 year of age and 1317 (14%) in children aged 1-2 years. Of the 285 839 episodes, 280 998 occurred in children 0-59 months old, of which 129 584 (46%) were 2-11 months of age and 152 730 (54%) were males.

**Conclusions:**

This data set could identify an improved specific, sensitive set of criteria for diagnosing clinical pneumonia and help identify sick children in need of referral to a higher level of care or a change of therapy. Field studies could be designed based on insights from PREPARE analyses to validate a potential revised pneumonia algorithm. The PREPARE methodology can also act as a model for disease database assembly.

Childhood pneumonia is one of the leading causes of mortality globally in children under the age of five, resulting in the death of around 800 000 children each year [1,2]. With under-five children as the most affected population group, particularly in low- and middle-income countries (LMICs), there is a clear need for effective interventions to reduce pneumonia-related mortality [1,3]. The World Health Organization (WHO) Integrated Management of Childhood Illnesses (IMCI), first developed in 1997, and updated in 2005 and 2014 (**Figure 1**), recommends that clinical findings should guide childhood pneumonia classification and antibiotic treatment decisions and treatment setting [4].

**Figure 1:**
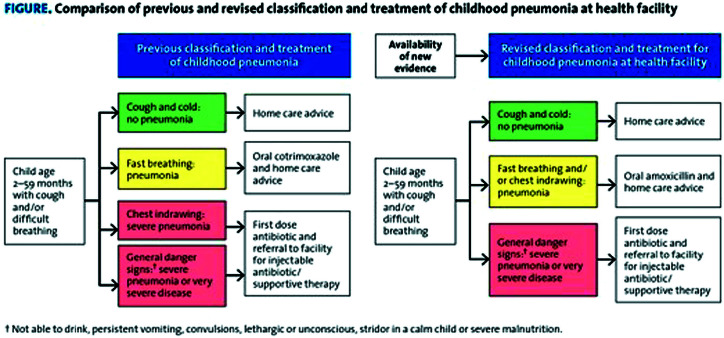
Classification of patients with pneumonia as per IMCI childhood pneumonia case management algorithm (2014).

The evidence-based, syndromic approach of the IMCI childhood pneumonia case management algorithm has been well-received due to its easy implementation by personnel with limited training in resource-poor settings [5]. The IMCI algorithm for pneumonia management has unknown sensitivity and specificity. The lower specificity of this algorithm for the diagnosis of pneumonia may contribute to unnecessary antibiotic use and greater referrals to hospitals [5,6]. Identification of signs and symptoms for improving diagnosis and outcome and decreasing treatment failure may allow for targeted antibiotic treatment and better care escalation decisions.

There are several potential reasons for the lower specificity of pneumonia signs. First, many children who meet the WHO pneumonia case definition have wheeze-associated viral infections [7-9]. Although the pneumonia case management algorithm recommends using rapid-acting bronchodilators in wheezing children before determining the need for antibiotic use, younger infants may not respond to bronchodilators, or a health facility may not have access to rapid-acting bronchodilators [4,10]. Therefore, some children may receive antibiotics when they do not require them. Second, fever is common in children with upper respiratory tract infections (URTI), acute lower respiratory infections (ALRI), or malaria [11-14]. Fever can increase the respiratory rate, meaning more children with a fever may be diagnosed with pneumonia [14,15]. Finally, hypoxemia, ie, oxygen saturation (SpO_2_ measurement) of less than 90% in peripheral arterial blood is an indicator of pneumonia severity and predictor of mortality in children [16,17]. WHO IMCI recommends the use of pulse oximetry (when available) to assess hypoxemia [4]. In facilities where pulse oximetry is unavailable, the clinical signs alone might miss hypoxemic children [18,19]. Pulse oximetry is now widely available and reliably used in health systems in low-resource settings [20-23]. The childhood pneumonia classification and treatment guidelines can benefit from better specificity.

In recent years, pneumonia studies conducted worldwide displayed wide variations in the case definitions for pneumonia [24-26]. There is no gold standard for the diagnosis of pneumonia, and different definitions have varying sensitivity and specificity, which affect results non-uniformly in analyses. Thus, there is a need to optimise the use of clinical signs for the diagnosis and prognosis of childhood pneumonia. The challenge posed by the different case definitions for pneumonia in studies could potentially be overcome, to some extent, with access to individual patient-level data (if the inclusion criteria in the studies are broadly similar). In recent years, epidemiological, vaccine, clinical, diagnostic, and treatment studies have been carried out, from which relevant data could be collated to potentially improve the clinical diagnosis, prognosis, and treatment of pneumonia.

We assembled a database from various collaborators on childhood pneumonia cases from both community-based and hospital-based settings in children aged 0-59 months. This Pneumonia Research Partnership to Assess WHO Recommendations (PREPARE) database included individual patient data from childhood pneumonia studies with similar case definitions, stratified by level of severity of pneumonia and study setting. We also included (where available) data from multiple clinical assessments over several days to permit multivariable analysis with robust endpoints, ie, death or clear signs of deterioration or severe hypoxemia. The primary aim of the PREPARE database was to answer specific questions about pneumonia diagnosis, prognosis, and treatment failure, and to inform the review of the current WHO standard case management guidelines for pneumonia. The three identified questions were: 1) Can we improve the clinical criteria for the diagnosis of pneumonia and thereby reduce unnecessary antibiotic treatment? 2) Can we identify those children who are at risk of an adverse outcome at the time of presentation and treat them appropriately and promptly? 3) Can we improve the identification of “true treatment failures” and deterioration, to appropriately manage them and reduce unnecessary treatment failures requiring a change in antibiotic therapy and/or hospitalisation? In this paper, we outline the methodology for collating the PREPARE database and provide a narrative description of this data set.

## METHODS

### Overview

**Figure 2:**
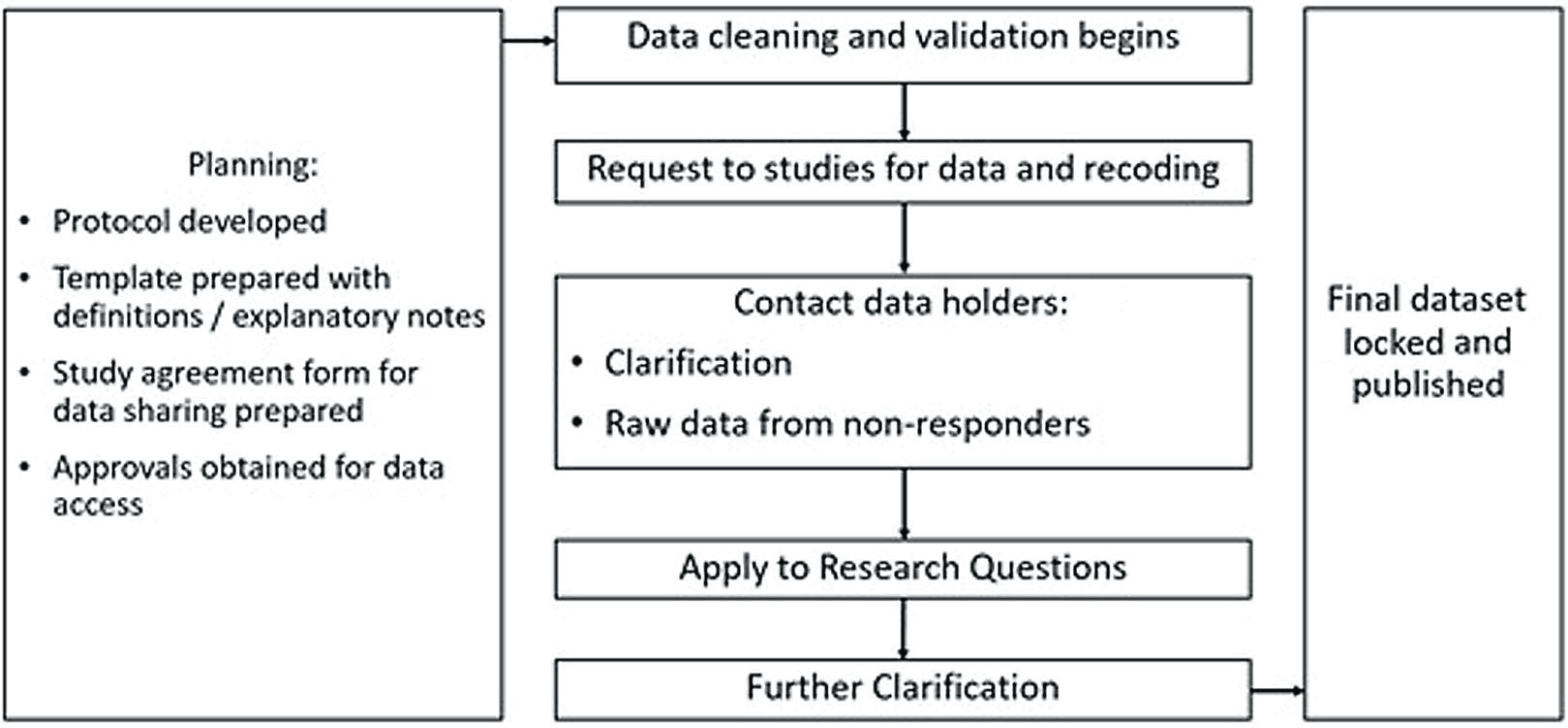
Protocol for planning, data collection, processing, and publication of PREPARE database.

We utilized the following steps in developing the PREPARE database. We spent a significant amount of time on planning, identifying various available databases that fulfilled our needs/requirements, reaching out to the investigators who had data, developing the conceptual, logical, and physical design, and constructing the database using Microsoft Excel. Subsequently, we acquired data and filled, cleaned, and prepared the database for analysis. The database is currently stored electronically at WHO headquarters. We followed an *a priori* protocol, as shown in **Figure 2**, to collate the final PREPARE database. Approvals to use data with the PREPARE investigator group were based on sharing the data with the WHO, free data sharing within the group, joint ownership of the PREPARE database, and agreement on a joint publication plan as guided by the WHO. Researchers outside the PREPARE study group can also access the data after sharing an outline of their proposed analysis using this data set, which will be approved by the WHO and PREPARE study group. The PREPARE study group will be acknowledged and given due recognition in manuscripts prepared because of various analyses. The study was conducted in full compliance with the Declaration of Helsinki. All individual studies had obtained approvals for their implementation. Local approvals were also obtained by individual sites to share the data with the WHO.

### Data collection design and process

The data collection protocol, variable collection templates, and data sharing agreement were developed collaboratively with the PREPARE group through an iterative process. Several sources informed the basis for variables included for collection described under information sources.

Draft data collection documents were compiled and sent to a smaller working group (including lead investigators, researchers at the University of Edinburgh, and WHO) for comment. The format of variables requested was discussed with statisticians at the University of Edinburgh, to ensure the data collected were in a suitable format for analysis to answer the PREPARE research questions.

The refined data collection documents comprised data collection templates for hospital and community-based studies, a study description template, a guide to data collection, variable dictionaries including definitions and coding of all variables, and a data-sharing agreement. Data collection templates were sent to study groups in a .csv format that could be read into all common statistical programmes.

Study groups returned data via a secure data repository held by the WHO. Researchers at the University of Edinburgh accessed data from this repository for data cleaning. The data cleaning process was undertaken in Microsoft Excel and SPSS, which involved the identification of missing or miscoded variables, and ensuring variables were correctly named and appropriate for collating. Study groups were contacted to clarify any discrepancies and missing data to ensure data were as complete as possible. Cleaned data were collated into two large data sets – one for hospital-based studies, and one for community-based studies, with each study allocated an identifier to aid analysis.

### Information sources

**Figure 3:**
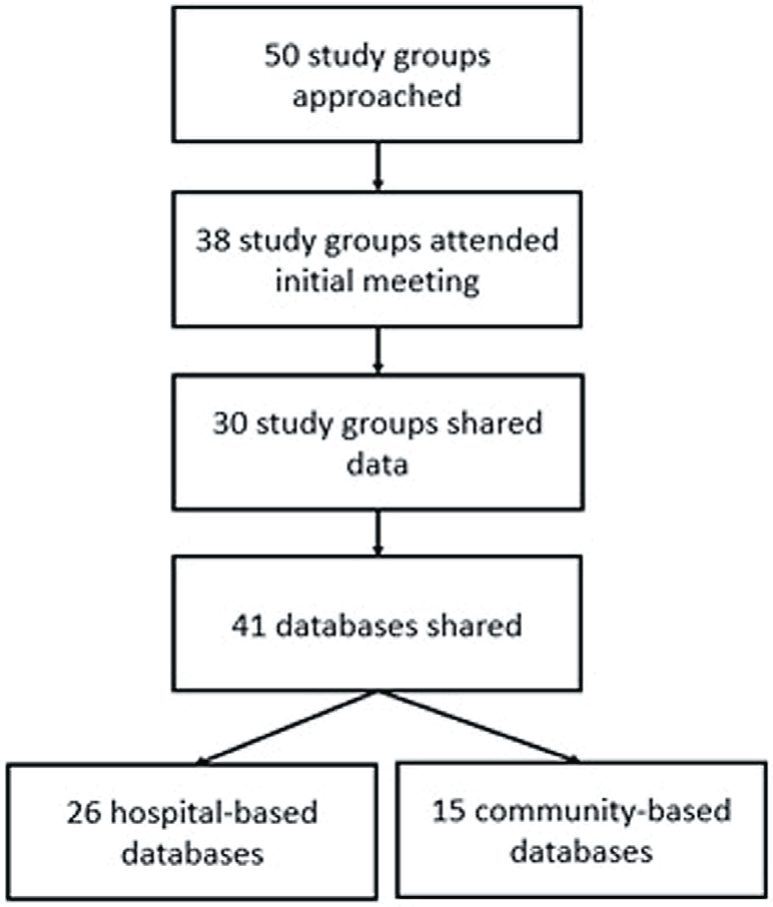
Process map to show how databases were collated.

We developed a list of research questions and data requirements to answer them, with a draft variable list and a data collection template, including a draft database of variables of interest. **Figure 3** details the methods, from contacting study groups to final database collation. We identified potential study groups from a list of contributors to a systematic review on the global burden of hospital admission for severe ALRI in children [27]. These study groups had earlier provided unpublished data for this systematic review and were therefore known to have high-quality data available for re-analysis. We added other collaborators based on the expert knowledge of scientists at WHO and other leading academic groups with knowledge of the childhood pneumonia research landscape. WHO hosted a meeting (15-17 October 2014) in Ferney Voltaire, France; 50 groups that had conducted pneumonia research – randomised controlled trials (including vaccines and antibiotics), observational studies, epidemiological studies in sick children/children with fever, and other intervention trials (eg, Vitamin B12) – from the early 2000s onwards were invited (Table S1 in the **Online Supplementary Document**). In this meeting, investigators from 38 groups participated and presented their data and agreed to share them for this exercise. Study sites were considered for inclusion in the WHO PREPARE database if they included data from control arms of vaccine/antibiotics trials, community-based cohorts, and hospital-based studies with clinical and epidemiological data collected. We sent two reminders to all non-responders before excluding them from our list. After the meeting, 30 research groups shared 41 databases; 26 from hospital-based studies and 15 from community-based studies. These data were from over 20 low- and middle-income countries, along with Australia and the United States of America. To align with the current IMCI protocols, only cases in children aged 0-59 months were included. There were no restrictions concerning the study setting.

### Study records

All data were received by the WHO, which commissioned the University of Edinburgh to collate and manage the de-identified data from all the studies. All identified studies were assessed for eligibility. The data collection process involved contact with lead investigators from the identified study groups. Two data collection templates were developed – one for longitudinal community-based studies (Table S2 in the **Online Supplementary Document**) and another for hospital-based studies (Table S3 in the **Online Supplementary Document**). A “Study description” template was developed to obtain an overview of each study and assess study comparability (Table S4 in the **Online Supplementary Document**). A variable dictionary containing detailed information about each variable, data formatting instructions and detailed case definitions were provided, to ensure data from each group used consistent units with comparable definitions wherever possible. The data received from investigator groups were cleaned and clarification was sought where necessary. Data cleaning was undertaken in Excel and SPSS. Researchers checked variables were correctly named and coded, identified outliers, erroneous values, and consistency of formatting across units and dates. To aid in this process, descriptive statistics such as frequency tables, histograms, medians, and ranges for each variable were obtained. All data were saved in .csv format to ensure merging was possible. We made multiple follow-ups to ensure completeness of study information sheets, to query abnormal values or dates, and to clarify categorical variables. If a child diagnosed with pneumonia was admitted to a hospital within 14 days of discharge, we assumed this was the continuation of the same episode; all admissions after 14 days of discharge were counted as separate episodes.

### Data items

In summary, we requested the sites to report the availability of more than 70 variables at the time of admission and on follow-up assessment by health care workers. We requested hospital-based studies to provide detailed individual patient data on demographic information and nutritional status related to the child, data on the previous hospitalisation for pneumonia in the past two weeks, history of symptoms relating to pneumonia (including current IMCI danger signs), findings on clinical examination at the time of admissions including vital signs, respiratory signs, and auscultatory findings. We also collected detailed data on chest radiograph findings and relevant laboratory investigations (**Table 1**). Additionally, we collected data on the diagnosis on admission, treatment (antibiotics and other interventions), clinical outcome, and diagnosis at the time the outcome was recorded. To understand clinical progression, we collected data on vital signs, pneumonia-specific and current IMCI danger signs, and oxygen saturation at 24-hour intervals from day 1 to day 14, where available.

**Table 1:** Summarised study descriptors

	Number of studies		
	**Community-based**	**Hospital-based**	**Total**
**Total number of studies**	15	26	41
**Geographic location**			
Latin America and the Caribbean	0	6	6
East Asia and the Pacific	0	6	6
Sub-Saharan Africa	11	5	16
South Asia	4	5	9
North America	0	3	3
Multicentre	0	1	1
**Location by World Bank income**			
High-income	0	4	4
Upper-middle	0	9	9
Lower-middle	12	7	19
Low-income	3	3	6
Not categorizable	0	3	3
**Hospital type**			
Tertiary (referral) hospital	NA	7	7
Secondary (district) hospital	NA	3	3
Both secondary and tertiary hospitals	NA	6	6
Hospitals with no ICU facilities	NA	1	1
Unclear hospital type	NA	9	9
**Pulse oximetry performed**			
Yes	1	22	23
No	14	4	18
**Chest radiography performed**			
Yes	1	23	24
No	14	3	17
**Bodyweight reported**			
Yes	2	20	22
No	13	6	19
**Length/height reported**			
Yes	1	14	15
No	14	12	26

NA – not applicable, ICU – intensive care unit

For community-based studies, we requested similar data on demographics, nutritional status, previous hospitalisation for pneumonia, history of symptoms relating to current illness (including current IMCI danger signs), findings on clinical examination, laboratory investigations and chest radiograph findings, clinical diagnosis, treatment given (including antibiotics), and outcome on each visit. Where the child was part of a cohort/clinical trial and could have multiple episodes of illness, these were all linked by using a unique identifier for a child.

### Data synthesis

We intended that the PREPARE data set complies with the FAIR (findable, accessible, interoperable, and reusable) data principles [28]. A study description sheet was completed by all study groups to allow us and future researchers to assess the comparability of study settings and study methods. Due to the nature of the data provided – from heterogeneous studies, designed and undertaken independently – comparability of data was at times limited and missing data an issue. All studies were assigned an identifier, which is attached to each case in the collated data sets, to ensure sub-group and sensitivity analyses can be undertaken. Additionally, for ease of data collation, we ensured all variable names were included in all study data sets, with missing data coded. We also complied with the recommendations for best practices for reporting the data sets included in individual patient data meta-analyses [29].

The PREPARE database includes collated data sets from hospital-based studies, collated data sets from community-based studies, their respective variable dictionaries and study descriptions, and a dictionary of case definitions used in included studies. We performed a narrative synthesis of the final data set. PREPARE database has been analysed to answer specific research questions, and while some of the results are published [20,30,31], a few other analyses are ongoing.

## RESULTS

Of the 41 data sets, six were in areas with a high pneumococcal conjugate vaccine (PCV) coverage (≥80%) and 14 in areas with some PCV coverage (<80%). In the remaining 21 studies, PCV was either not introduced in the study area or coverage was unknown at the time of data collection. The included studies ranged from cohort studies on febrile illnesses in children and childhood pneumonia to vaccine and other intervention trials (eg, antibiotics, Vitamin B12). Thus, only the subset of children who had pneumonia were included in the data set.

**Figure 4:**
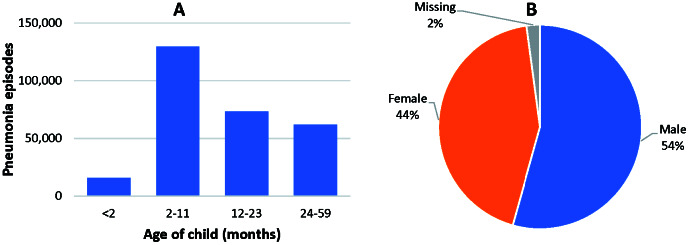
**Panel A:** Pneumonia episodes in all settings, by age category in children 0-59 months of age. **Panel B:** Pneumonia episodes in all settings, by sex in children 0-59 months of age.

The database included a total of 285 839 pneumonia episodes (244 323 in the hospital and 41 516 in the community). The outcome was reported in 263 487 episodes, including 9185 deaths in children (**Table 2**). Of the reported 285 839 episodes, 280 997 occurred in children 0-59 months old, age was missing in 4815 episodes, and 26 episodes occurred in children aged over 59 months. As shown in **Figure 4**, Panel A, of the 280 998 episodes in children 0-59 months old, 129 584 (46%) occurred in children 2-11 months. **Figure 4**, Panel B 152 730 (54%) pneumonia episodes were in males. An analysis of the mortality data showed that 74% (6836/9185) of all pneumonia-related deaths occurred in the age group <1 year, and an additional 14% (1317/9185) occurred in the second year of life.

**Table 2:** Number of pneumonia episodes and deaths, with age and sex breakdown (n = 285 839)

	Number of pneumonia episodes		
	**Community-based**	**Hospital-based**	**Total**
**Total number of episodes**	41 516	244 323	285 839
**Sample size per study –** Median (IQR)	1332 (900-4410)	1509 (817-5043)	NA
**Age category (months)**			
<2	1186 (2.9%)	14670 (6.0%)	15 856 (5.6%)
2-11	15 996 (38.5%)	113 588 (46.5%)	129 584 (45.3%)
12-23	12 641 (30.4%)	60 855 (24.9%)	73 496 (25.7%)
24-59	10 919 (26.3%)	51 143 (20.9%)	62 062 (21.7%)
Missing*	774 (1.9%)	4067 (1.7%)	4841 (1.7%)
**Sex**			
Male	20 281 (48.9%)	135 042 (55.3%)	15 5323 (54.3%)
Female	18 774 (45.2%)	105 620 (43.2%)	12 4394 (43.5%)
Missing	2461 (5.9%)	3661 (1.5%)	6122 (2.1%)
	**Number of pneumonia-related deaths**		
**Total number of pneumonia-related deaths**	37	9148	9185
**Pneumonia deaths –** Median (IQR)	2 (0-6)	17 (2-132)	NA
**Age category (months)**			
<2	3 (8.1%)	733 (8.0%)	736 (8.0%)
2-11	20 (54.1%)	6080 (66.5%)	6100 (66.4%)
12-23	6 (16.2%)	1311 (14.3%)	1317 (14.3%)
24-59	7 (18.9%)	696 (7.6%)	703 (7.6%)
Missing*	1 (2.7%)	328 (3.6%)	329 (3.6%)

IQR – interquartile range, NA – not applicable

*Including 26 children with age >5 y.

### Hospital-based studies

**Figure 5:**
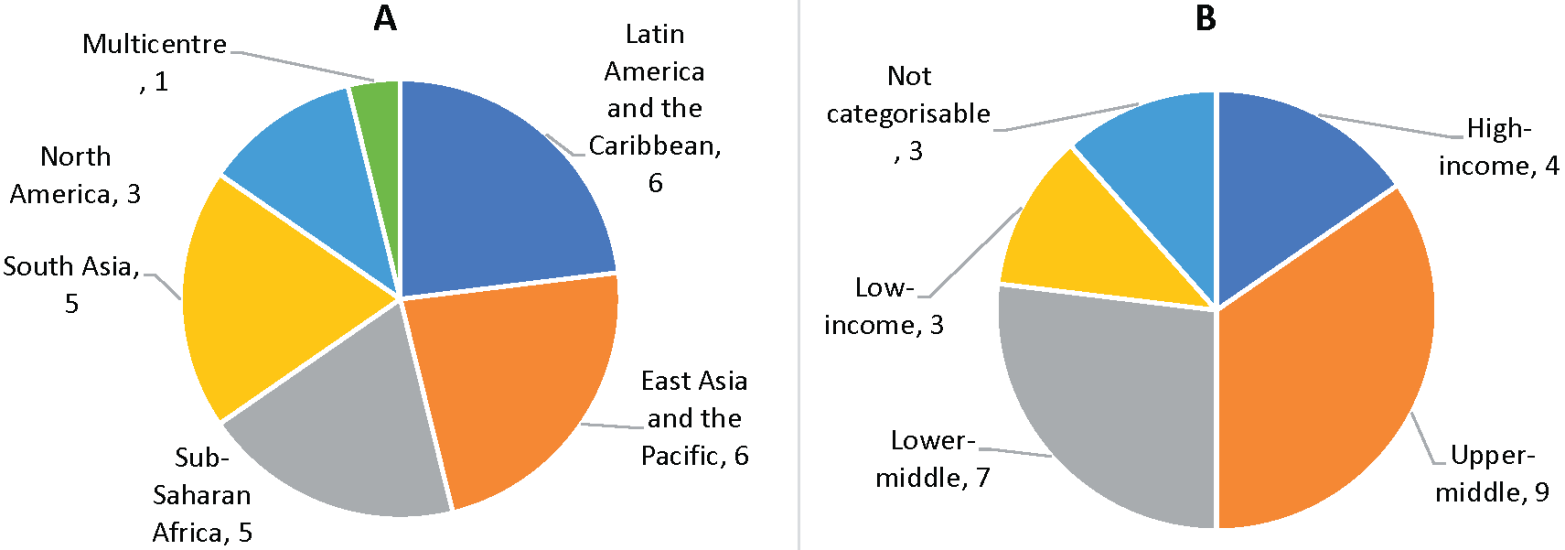
**Panel A**: Hospital-based studies by geographical location. **Panel B:** Number of hospital-based studies by location income (World Bank 2021 Income Classification) [30].

**Figure 5**, Panel A shows the geographical spread of the 26 studies amongst regions of the world. **Figure 5****,** Panel B shows the study setting by the income of the country; the majority of studies were in upper-middle (n = 9) and lower-middle income countries (n = 7) [32]. The median sample size across hospital-based studies was 1509 (interquartile range (IQR) = 817-5043), and the median number of pneumonia deaths was 17 (IQR = 2-132). Seven of 26 studies were based only in tertiary (referral) hospitals, three studies were based only in secondary (district) hospitals, and six included both secondary and tertiary hospitals. Nine studies were not categorizable by hospital type.

There were 9148 pneumonia-related deaths among 244 323 episodes (3.74%). Of these, 733 deaths were in children <2 months, 6080 were in children 2-11 months, 1311 were in children 12-23 months, 696 were in children 24-59 months of age, and the remaining 328 were in children of unknown age.

A total of 47263 chest radiographs were taken, of which 24901 (53%) children had radiological pneumonia. A chest radiograph was available for 1228 child deaths, of which 429 had radiological pneumonia.

### Community-based studies

Out of 15 community-based studies, 11 were based in South Asia, four were based in Sub-Saharan Africa, and all studies were in low-income (three studies) or lower-middle-income countries (12 studies) [32]. The median sample size was 1322 (IQR = 900-4410), and the median number of pneumonia deaths was 2 (IQR = 0-6).

In the community-based studies, there were 37 pneumonia deaths in total, with 54% (20/37) occurring in children 2-11 months of age. A total of 15256 chest radiographs were taken, of which 9491 (62%) children had radiological pneumonia. A chest radiograph was available for 13 child deaths, of which six had radiological pneumonia.

## DISCUSSION

This database is the first global database on childhood pneumonia, incorporating a total of 285 839 pneumonia cases in children less than 5, from both hospital-based and community-based studies. Combining individual cases from 41 studies conducted in diverse settings is highly novel and leverages existing research for greater impact on literature. A major outcome of the PREPARE project was the creation of a clean database from 41 data sets coded in a common format with related metadata such as study descriptors, variable dictionaries, and a list of definitions. This database is available to interested researchers outside the PREPARE investigators through WHO, to explore study questions related to pneumonia diagnosis, prognosis, and treatment failure and to confirm findings from data from individual study sites. Most pneumonia cases and pneumonia-related deaths were from hospital-based sites, likely due to the greater availability of clinical variables in a hospital setting. Community-based settings recorded primarily respiratory rate observation, pulse oximetry measurements, first-line antibiotic choice and seldom involved chest radiography.

The analysis from the PREPARE data set would be useful if we could identify criteria that could enhance the early identification of the severity of illness, assist in referral decisions, or allocate cases to the appropriate levels of the health care system where the pneumonia case could best be managed. Improving the prognostic indicators to identify deterioration more appropriately, such as appropriate identification and management of hypoxaemia, could reduce deaths and disease complications. It could lead to not changing antibiotics unnecessarily in children who are not at higher risk, limiting the use of a broader spectrum and more expensive second-line or rescue therapy, thus rationalizing antibiotic use and less pressure on antimicrobial resistance. It could lessen hospitalisations, resulting in less pressure on hospital staff, reduced injectable antibiotic use, risk of injection-based adverse effects, and reduced hospital infections. It could potentially lower the health systems costs’ resulting in more efficient programming by using limited resources in LMICs by focusing on pneumonia management strategies where needed. Additionally, it could increase convenience for the families and reduce family costs due to the reduction of unnecessary referrals.

The PREPARE data set reported that 92% of deaths occurred in children under two years of age, which is substantially greater than the 81% reported by Walker et al. [33]. Given that pneumonia mortality is increasingly found in the <2-year age group and given the concerns about overuse of antibiotics, these findings argue for consideration to be given to prioritising this age group in future pneumonia control programme activities and in the field studies to evaluate new case management algorithms. This change would focus case management efforts on the highest mortality group and substantially reduce antibiotic use.

### Strengths

We were able to gather data on precise case definitions used in each study and detailed clinical data at an individual patient level on several variables of interest to understand status at presentation, progression of illness and outcome.

Furthermore, we were able to adequately consider the study context (under-5 mortality rate, the prevalence of wheeze, etc.) due to the detailed reliable individual patient-level data in the PREPARE database. This will allow for sub-analyses of the data set and will aid and guide power calculations for future field-based studies.

### Limitations

As almost all included studies used existing WHO IMCI case definitions (cough, difficulty breathing, and respiratory rate thresholds) as part of their inclusion criteria, this data set cannot be used to validate current WHO IMCI pneumonia case definitions. Also, it is important to note that included studies had not generally been designed to consider the validity of clinical signs for the diagnosis of clinical pneumonia by first-level health workers. Hence, this database is not able to estimate or model the likely performance of these combinations of signs in real-life LMIC settings on an outpatient basis. Additionally, some regions are underrepresented in this data set, limiting external validity. Lastly, the timing of presentation to study sites could be different in various studies and has not been considered. Further information on study design should be collected as studies ranged from uncontrolled studies with passive or no surveillance to highly controlled studies involving active case-finding. Finally, as all studies had been designed and data collected independently, there was variation in which variables had been collected and how they had been collected. This led to missing data across most variables.

This data set can potentially identify an improved specific and sensitive set of criteria to diagnose clinical pneumonia and identify sick children in need of a referral, change of therapy, or shift to a higher level of care. The analyses could assist the WHO to review its pneumonia management protocol and guide the setting-specific investigation of applicability. Field studies could be designed based on insights from PREPARE analyses to validate a revised pneumonia management algorithm. The PREPARE methodology can also act as a model for disease database assembly.

## CONCLUSIONS

In the absence of one central database, it is difficult to investigate case management algorithms while ensuring the sample size is sufficient and results are externally valid. The database may guide insight into research questions, tackling knowledge gaps in the diagnosis, prognosis and treatment of pneumonia, and guide future studies to be sufficiently powered to allow for meaningful results with clinical application.

## Additional material


Online Supplementary Document
