# Supplementary material for: Assembling a global database of child pneumonia studies to inform WHO pneumonia management algorithm: Methodology and applications
Source: J Glob Health. 2022 Dec 29;12:04075. doi: 10.7189/jogh.12.04075 (PMC9798037; doi:10.7189/jogh.12.04075)
Supplement: Online Supplementary Document [file jogh-12-04075-s001.pdf]

ONLINE SUPPLEMENTARY DOCUMENT

Title: Assembling a global database of child pneumonia studies to inform WHO pneumonia management algorithm: methodology and applications

Helena Martin, Jennifer Falconer, Emmanuel Addo-Yobo, Satinder Aneja, Luis Martinez Arroyo, Rai Asghar, Shally Awasthi, Salem Banajeh, Abdul Bari, Sudha Basnet, Ashish Bavdekar, Nita Bhandari, Shinjini Bhatnagar, Zulfiqar A. Bhutta, Abdullah Brooks, Mandeep Chadha, Noel Chisaka, Monidarin Chou, Alexey W. Clara, Tim Colbourn, Clare Cutland, Valérie D'Acremont, Marcela Echavarria, Angela Gentile, Brad Gessner, Christopher J. Gregory, Tabish Hazir, Patricia L. Hibberd, Siddhivinayak Hirve, Shubhada Hooli, Imran Iqbal, Prakash Jeena, Cissy B. Kartasasmita, Carina King, Romina Libster, Rakesh Lodha, Juan M. Lozano, Marilla Lucero, Norman Lufesi, William B. MacLeod, Shabir Ahmed Madhi, Joseph L. Mathew, Irene Maulen-Radovan, Eric D. McCollum, Greta Mino, Charles Mwansambo, Mark I. Neuman, Ngoc Tuong Vy Nguyen, Marta C. Nunes, Pagbajabyn Nymadawa, Kerry-Ann F. O'Grady, Jean-William Pape, Glaucia Paranhos-Baccala, Archana Patel, Valentina Sanchez Picot, Mala RakotoAndrianarivelo, Zeba Rasmussen, Vanessa Rouzier, Graciela Russomando, Raul O.
Ruvinsky, Salim Sadruddin, Samir K. Saha, Mathuram Santosham, Sunit Singhi, Sajid Soofi, Tor A. Strand, Mariam Sylla, Somsak Thamthitiwat, Donald M. Thea, Claudia Turner, Philippe Vanhems, Nitya Wadhwa, Jianwei Wang, Syed MA. Zaman, Harry Campbell, Harish Nair, Shamim Ahmad Qazi, Yasir Bin Nisar on behalf of the World Health Organization PREPARE study group

Table S1. Timetable of project activities

Date and Description of Event	
October 2014	PREPARE project meeting, Geneva
January 2015 -	Protocol developed among project subgroup<br>Template prepared with definitions / explanatory notes<br>Study agreement form for data sharing prepared Approvals obtained for data access
April 2015	Data sharing agreement finalised; request sent to study investigators for data and data recoding
October 2015	Initial tranche of data received. Data received by WHO with copy shared with the University of Edinburgh via access to a secure server site. Data cleaning and validation began; clarifications were sought from all studies and a final set of reminders was sent to non-responders
February 2016	Data collection completed (data received from 31 study groups); the first round of data cleaning completed
March 2016	Inspection of data suitable for analysis for research questions 1-4 leading to further round of data requests with a final deadline of May to receive data
April 2016	Data analysis started on available data; involved further rounds of data clarifications and re-formatting
June 2016	Final dataset locked for analysis; analysis of the full dataset began
September 2016	PREPARE meeting in Geneva to discuss progress and interim results completed to that date

The data clarification/cleaning exercise was extensive and required contact with all study sites. Multiple follow-up/queries (up to 15 pages of clarifications) were required in most studies. The majority of the queries concerned:<br>• missing study information sheets,<br>• abnormal values in requested variables,<br>• dates in strange formats,<br>• categorical variables with codes that did not match our variable dictionary,<br>• new variables that had been added, but no explanation was provided<br>• querying missing data/data completeness<br>• re-scoring to a more standard approach
For each specific research question data were re-formatted e.g.<br>• A list of variables and definitions for each PREPARE question was created following the analysis approach from the PREPARE analysis subgroup<br>• For each specific research question data were cleaned and re-formatted e.g.<br>• deleting non-applicable variables,<br>• re-coding from continuous variables to categorical variables depending on the question,<br>• creating new variables from the information provided<br><br>Information was obtained to get more clarity on:<br>• definitions, inclusion and exclusion criteria and how CXRs were reported<br>• which datasets were suitable to answer specific research questions and what predictor and outcome data were available<br><br>Approvals to use data with the PREPARE investigator group were based on:<br>• free data sharing within the group no data to be placed in the public domain at this stage<br>• joint ownership of the PREPARE database<br>• agreement on a joint publication plan as guided by WHO

Table S2. Variable Dictionary: Data Collection for Community-based Studies

	Type	Explanation	Format of return
Demographic Information	descriptive	Episode ID - one episode of pneumonia/bronchiolitis should be entered per row.	Free text - please enter this however you recorded it.
	descriptive	Your ID code for the child	Free text - please enter this however you recorded it.
	Descriptive	Location of study site where the child was assessed	Free text – Please enter the Location of the study site.
	date	Date of birth of the child	DD/MM/YYYY
	continuous	Age of child	Days
	categorical	Sex of child	0 (Male); 1 (Female); 2 (Don't Know)
	categorical	Maternal HIV status	0 (Negative); 1 (Positive); 2 (Don't Know)
General Medical History	categorical	Any previous hospitalisations in the past 2 weeks?	0 (No); 1 (Yes); 2 (Don't Know); 999 (Not Applicable)
	Categorical	Was this previous hospitalisation for pneumonia?	0 (No); 1 (Yes); 2 (Don't Know); 999 (Not Applicable)
History of Current Illness	date	Date of onset of current illness	DD/MM/YYYY
	categorical	History of Cough within the past 14 days	0 (No); 1 (Yes); 2 (Don't Know); 999 (Not Applicable)
	continuous	Duration of cough	Days
	categorical	History of difficulty breathing within the past 14 days	0 (No); 1 (Yes); 2 (Don't Know); 999 (Not Applicable)
	continuous	Duration of difficulty breathing	Days
	categorical	History of fever (as reported by caregiver) within the past 14 days	0 (No); 1 (Yes); 2 (Don't Know); 999 (Not Applicable)
	continuous	Duration of fever (as reported by caregiver)	Days
	categorical	History of inability to drink or breastfeed within the past 14 days	0 (No); 1 (Yes); 2 (Don't Know); 999 (Not Applicable)
	continuous	Duration of inability to drink or breastfeed	Days
	categorical	History of convulsions/seizures within the past 14 days	0 (No); 1 (Yes); 2 (Don't Know); 999 (Not Applicable)
	continuous	Duration of convulsions/seizure	Days

	categorical	History of vomiting everything within the past 14 days	0 (No); 1 (Yes); 2 (Don't Know); 999 (Not Applicable)
	continuous	Duration of vomiting everything	Days
	categorical	History of decreased level of consciousness within past 14 days. This includes a history of being dull/unresponsive, abnormally sleepy, difficult to wake, lethargy and prostration.	0 (No); 1 (Yes); 2 (Don't Know); 999 (Not Applicable)
	continuous	Duration of decreased level of consciousness	Days
	categorical	Use of antibiotics within the past 14 days	0 (No); 1 (Yes); 2 (Don't Know); 999 (Not Applicable)
Examinations in Outpatient Setting – Exam 1, Exam 2, Exam 3	categorical	Were any examinations done on the child before hospitalisation? E.g. in an outpatient department or clinic, or at a home visit.	0 (No); 1 (Yes); 2 (Don't Know)
	categorical	Location of the first examination done in the outpatient setting	1 (home visit); 2 (clinic); 3 (outpatient department); 4 (other)
	descriptive	If 'other' is listed for V1PH_LOCAT, please specify it here	Free text
	date	Date of examination in an outpatient setting	DD/MM/YYYY
	continuous	Respiratory rate (measured when child calm) at the examination in an outpatient setting	Breaths/Minute
	continuous	The temperature at the examination in an outpatient setting	Degrees Celsius
	categorical	The site at which temperature was measured	1 (oral); 2 (axillary); 3 (rectal); 4 (tympanic); 5 (Don't know)
	categorical	Cough at the examination in an outpatient setting	0 (No); 1 (Yes); 2 (Don't Know); 999 (Not Applicable)
	categorical	Runny nose at the examination in an outpatient setting	0 (No); 1 (Yes); 2 (Don't Know); 999 (Not Applicable)
	categorical	Difficulty breathing at the examination in an outpatient setting	0 (No); 1 (Yes); 2 (Don't Know); 999 (Not Applicable)
	categorical	Apnoea at the examination in an outpatient setting	0 (No); 1 (Yes); 2 (Don't Know); 999 (Not Applicable)
	categorical	Audible wheeze at the examination in an outpatient setting	0 (No); 1 (Yes); 2 (Don't Know); 999 (Not Applicable)

	categorical	Audible stridor at the examination in an outpatient setting	0 (No); 1 (Yes); 2 (Don't Know); 999 (Not Applicable)
	categorical	Audible grunting at the examination in an outpatient setting	0 (No); 1 (Yes); 2 (Don't Know); 999 (Not Applicable)
	categorical	Nasal flaring at the examination in an outpatient setting	0 (No); 1 (Yes); 2 (Don't Know); 999 (Not Applicable)

	categorical	Lower chest wall indrawing (subcostal retractions) at the examination in an outpatient setting	0 (No); 1 (Yes); 2 (Don't Know); 999 (Not Applicable)
	categorical	Inability to drink at the examination in an outpatient setting	0 (No); 1 (Yes); 2 (Don't Know); 999 (Not Applicable)
	categorical	Difficulty breastfeeding at the examination in an outpatient setting	0 (No); 1 (Yes); 2 (Don't Know); 999 (Not Applicable)
	categorical	Convulsions/seizures at the examination in an outpatient setting	0 (No); 1 (Yes); 2 (Don't Know); 999 (Not Applicable)
	categorical	Cyanosis at the examination in an outpatient setting	0 (No); 1 (Yes); 2 (Don't Know); 999 (Not Applicable)
	categorical	Head nodding/bobbing at the examination in an outpatient setting	0 (No); 1 (Yes); 2 (Don't Know); 999 (Not Applicable)
	categorical	Decreased consciousness at the examination in an outpatient setting. Includes dull/unresponsive, abnormally sleepy, difficult to wake, lethargy and prostration.	0 (No); 1 (Yes); 2 (Don't Know); 999 (Not Applicable)
	categorical	Irritable/agitated at the examination in an outpatient setting	0 (No); 1 (Yes); 2 (Don't Know); 999 (Not Applicable)
	continuous	Oxygen saturation at the examination in an outpatient setting	%
	continuous	Heart rate at the examination in an outpatient setting	Beats/Minute
	categorical	What diagnosis was given at the examination in an outpatient setting	1 (Pneumonia - all severities); 2 (Cough or cold); 3 (Bronchiolitis); 4 (Bronchitis); 5 (Wheezy Bronchitis); 6 (Asthma); 7 (Other - specify below)
	descriptive	If 'other' is listed under 'V1PH_DIAG', please specify it here	Free text
	categorical	How was the diagnosis made at the examination in an outpatient setting	1 (physician diagnosis); 2 (Healthworker diagnosis)

V1PH_CXR			
Chest Xray findings in the outpatient setting – Examinations 1,2,3	categorical	A chest X-ray was taken when the child was seen in the outpatient setting seen for the first time during the illness episode.	0 (No); 1 (Yes); 2 (Don't Know)
	date	The date on which the child's chest x-ray was taken in an outpatient setting	DD/MM/YYYY
	categorical	Chest X-ray quality of CXR in an outpatient setting	0 (Uninterpretable); 1 (Suboptimal); 2 (Adequate)
	categorical	Number of readers interpreting the outpatient chest x-ray	1 (1 reader); 2 (2 readers); 3 (3 readers); 4 (2 readers and 1 arbiter)
	categorical	Outpatient Chest x-ray findings meeting Primary-Endpoint Criteria (see Definitions document)	0 (No); 1 (Yes); 2 (Don't Know)
	categorical	Outpatient Chest x-ray findings showing lobar pneumonia/consolidation	0 (No); 1 (Yes); 2 (Don't Know)
	categorical	Pleural effusion present on chest x-ray taken in an outpatient setting	0 (No); 1 (Yes); 2 (Don't Know)
	categorical	Abscess present on chest x-ray taken in an outpatient setting	0 (No); 1 (Yes); 2 (Don't Know)
	categorical	Other abnormal findings are present on chest x-ray taken in an outpatient setting. E.g. atelectasis/pneumatocele/pneumothorax	0 (No); 1 (Yes); 2 (Don't Know)
	categorical	Any other infiltrate present on chest x-ray taken in an outpatient setting	0 (No); 1 (Yes); 2 (Don't Know)
	categorical	Outpatient Chest X-ray normal	0 (No); 1 (Yes); 2 (Don't Know)
The outcome of Outpatient Assessment – Exam 1, 2, 3	categorical	The outcome of the first examination done in an outpatient setting	1. (Child referred to hospital); 2. (Child sent home with injectable antibiotics); 3. (child sent home on oral antibiotics); 4 (child sent home with symptomatic management - but no antibiotics given); 5 (Child recovered)
	date	If ‘child recovered’ is given for V1PH_OUTCM, please specify the date of recovery here (if available)	DD/MM/YYYY
Examination at the time of Hospital	categorical	Child hospitalized	0 (No); 1 (Yes); 2 (Don't Know); 999

Admission			(Not Applicable)
	date	Date of hospital admission	DD/MM/YYYY
	Categorical	Diagnosis at the hospital admission. Please specify.	1 (Pneumonia - all severities); 2 (Bronchiolitis); 3 (Bronchitis); 4 (Wheezy Bronchitis); 5 (Asthma); 6 (Other - specify below)
	descriptive	If 'other' is listed as a hospital admission diagnosis in DIAG_ADM, please specify the diagnosis here.	Free text
	Categorical	Diagnosis at discharge, or final diagnosis. Please specify.	1 (Pneumonia - all severities); 2 (Bronchiolitis); 3 (Bronchitis); 4 (Wheezy Bronchitis); 5 (Asthma); 6 (Other - specify below)
	descriptive	If 'other' is listed as discharge diagnosis in DIAG_DIS, please specify the diagnosis here.	Free text

	continuous	Duration of the hospital stay	Days
Vital Signs at Hospital Admission	continuous	Respiratory Rate (measurement 1) on admission to the hospital (measured when child calm)	Breaths/Minute
	continuous	Respiratory Rate (measurement 2, if taken) on admission to the hospital (measured when child calm)	Breaths/Minute
	continuous	The temperature on admission to the hospital	Degrees Celsius
	categorical	The site at which temperature was measured	1 (oral); 2 (axillary); 3 (rectal); 4 (tympanic); 5 (Don't know); 999 (Not Applicable)
	continuous	Heart Rate on admission to the hospital	Beats/Minute
Respiratory Signs at Hospital Admission	categorical	Cough on admission to the hospital	0 (No); 1 (Yes); 2 (Don't Know); 999 (Not Applicable)
	categorical	Difficulty breathing on admission to the hospital	0 (No); 1 (Yes); 2 (Don't Know); 999 (Not Applicable)
	categorical	Apnoea on admission to the hospital	0 (No); 1 (Yes); 2 (Don't Know); 999 (Not Applicable)

	categorical	Audible wheeze on admission to the hospital	0 (No); 1 (Yes); 2 (Don't Know); 999 (Not Applicable)
	categorical	Audible stridor on admission to the hospital	0 (No); 1 (Yes); 2 (Don't Know); 999 (Not Applicable)
	categorical	Audible grunting on admission to the hospital	0 (No); 1 (Yes); 2 (Don't Know); 999 (Not Applicable)
	categorical	Nasal flaring on admission to the hospital	0 (No); 1 (Yes); 2 (Don't Know); 999 (Not Applicable)
	categorical	Presence of lower chest indrawing/subcostal retractions on admission to the hospital	0 (No); 1 (Yes); 2 (Don't Know); 999 (Not Applicable)
	categorical	Presence of intercostal indrawing/intercostal retractions on admission to the hospital	0 (No); 1 (Yes); 2 (Don't Know); 999 (Not Applicable)
	categorical	The runny nose on admission to the hospital	0 (No); 1 (Yes); 2 (Don't Know); 999 (Not Applicable)
	Categorical	Ear discharge on admission to the hospital	0 (No); 1 (Yes); 2 (Don't Know); 999 (Not Applicable)
Auscultatory Signs at Hospital Admission	categorical	Bronchial breathing on auscultation on admission to the hospital	0 (No); 1 (Yes); 2 (Don't Know); 999 (Not Applicable)
	categorical	Crepitations/rales/crackles present on auscultation on admission to the hospital	0 (No); 1 (Yes); 2 (Don't Know); 999 (Not Applicable)
	categorical	Wheeze/ rhonchi on auscultation on admission to the hospital	0 (No); 1 (Yes); 2 (Don't Know); 999 (Not Applicable)
Danger Signs at Hospital Admission	categorical	Inability to drink or feed (including breastfeeding) on admission to the hospital	0 (No); 1 (Yes); 2 (Don't Know); 999 (Not Applicable)
	categorical	Convulsions/seizures on admission to the hospital	0 (No); 1 (Yes); 2 (Don't Know); 999 (Not Applicable)
	categorical	Cyanosis on admission to the hospital	0 (No); 1 (Yes); 2 (Don't Know); 999 (Not Applicable)
	categorical	Head nodding/bobbing on admission to the hospital	0 (No); 1 (Yes); 2 (Don't Know); 999 (Not Applicable)

	categorical	Decreased consciousness on admission to the hospital. Includes dull/unresponsive, abnormally sleepy, difficult to wake, lethargy and prostration.	0 (No); 1 (Yes); 2 (Don't Know); 999 (Not Applicable)
	categorical	The decreased activity level upon admission to the hospital	0 (No); 1 (Yes); 2 (Don't Know); 999 (Not Applicable)
	categorical	Irritable/agitated on admission to the hospital	0 (No); 1 (Yes); 2 (Don't Know); 999 (Not Applicable)
General Examination at Hospital Admission	continuous	Weight on admission to the hospital	kg
	continuous	Height on admission to the hospital	cm
	continuous	Mid Upper Arm Circumference on admission to the hospital	cm
	continuous	Oxygen saturation on admission to the hospital	%
	Categorical	Was oxygen saturation measured on room air or supplementary oxygen	1 (Room Air); 2 (Supplementary Oxygen); 3 (Don't Know); 999 (Not Applicable)
Chest X-ray Findings at Hospital Admission	categorical	Chest X-ray was taken in an inpatient setting	0 (No); 1 (Yes); 2 (Don't Know)
	date	The date on which the child's chest x-ray was taken	DD/MM/YYYY
	categorical	Chest X-ray quality	0 (Uninterpretable); 1 (Suboptimal); 2 (Adequate)
	categorical	Number of readers interpreting the chest x-ray	1 (1 reader); 2 (2 readers); 3 (3 readers); 4 (2 readers and 1 arbiter)
	categorical	Chest x-ray findings meeting Primary-Endpoint Criteria (see Definitions document)	0 (No); 1 (Yes); 2 (Don't Know)
	categorical	Chest x-ray findings showing lobar pneumonia/consolidation	0 (No); 1 (Yes); 2 (Don't Know)
	categorical	Pleural effusion present on chest x-ray	0 (No); 1 (Yes); 2 (Don't Know)
	categorical	Abscess present on chest x-ray	0 (No); 1 (Yes); 2 (Don't Know)
	categorical	Other abnormal findings were present on the chest x-ray. e.g., atelectasis/pneumatocele/pneumothorax	0 (No); 1 (Yes); 2 (Don't Know)
	categorical	Any other infiltrate present on chest x-ray	0 (No); 1 (Yes); 2 (Don't Know)
	categorical	Chest X-ray normal	0 (No); 1 (Yes); 2 (Don't Know)

Vaccination Status	categorical	Number of doses of pentavalent[1] vaccination child has received	0 (0 doses); 1 (1 dose); 2 (2 doses); 3 (3 doses); 4 (doses not known; vaccination up to date, as verified by vaccination card); 999 (Not Applicable)
	categorical	Number of doses of PCV vaccination child has received	0 (0 doses); 1 (1 dose); 2 (2 doses); 3 (3 doses); 4 (doses not known; vaccination up to date, as verified by vaccination card); 999 (Not Applicable)
	categorical	Number of doses of Hib vaccination child has received	0 (0 doses); 1 (1 dose); 2 (2 doses); 3 (3 doses); 4 (doses not known; vaccination up to date, as verified by vaccination card); 999 (Not Applicable)
	categorical	Number of doses of DPT (Diphtheria, Pertussis, Tetanus) vaccination child has received	0 (0 doses); 1 (1 dose); 2 (2 doses); 3 (3 doses); 4 (doses not known; vaccination up to date, as verified by vaccination card); 999 (Not Applicable)
	categorical	Number of doses of Measles vaccination child has received	0 (0 doses); 1 (1 dose); 2 (2 doses); 3 (doses not known; vaccination up to date, as verified by vaccination card); 999 (Not Applicable)
Treatment	categorical	Bronchodilator challenge received (see Definitions document)	0 (No); 1 (Yes); 2 (Don't Know); 999 (Not Applicable)
	categorical	Result of bronchodilator challenge - fail or success	0 (Fail); 1 (Success); 2 (Don't Know); 999 (Not Applicable)
	categorical	Antibiotics were given to the child	0 (No); 1 (Yes); 2 (Don't Know)
	Continuous	Duration of antibiotic treatment	Days

[1] The pentavalent vaccine includes DPT (Diphtheria, Pertussis, Tetanus), Hepatitis B, and Hib vaccines.

	categorical	Was there a change of antibiotic treatment given to the child(s)	0 (No); 1 (Yes); 2 (Don't Know); 999 (Not Applicable)
	date	Date of change of antibiotic(s)	DD/MM/YYYY
	categorical	Reason for change of antibiotic(s)	0 (persistence of signs); 1 (clinical deterioration - development of new danger signs); 2 (Clinical deterioration - fall in oxygen saturation); 3 (Failure to improve); 4 (Other - please specify below); 5 (Don't Know); 999 (Not Applicable)
	descriptive	If 'other' is listed as the reason for the change in ABX_CHREAS, please specify the reason here	Free text
	categorical	Was the course of antibiotics completed successfully?	0 (No); 1 (Yes); 2 (Don't Know); 999 (Not Applicable)
	categorical	Use of bronchodilator during illness	0 (No); 1 (Yes); 2 (Don't Know); 999 (Not Applicable)
	categorical	Supplemental oxygen was given	0 (No); 1 (Yes); 2 (Don't Know); 999 (Not Applicable)
	date	Date child is first given supplemental oxygen	DD/MM/YYYY
	continuous	Duration of supplemental oxygen use	Hours
	categorical	ICU/intensive admission or care	0 (No); 1 (Yes); 2 (Don't Know); 999 (Not Applicable)
	categorical	Need for ventilation (any - manual or mechanical), regardless of availability. Includes children who needed ventilation but were not given it.	0 (No); 1 (Yes); 2 (Don't Know); 999 (Not Applicable)
	categorical	Manual ventilation given	0 (No); 1 (Yes); 2 (Don't Know); 999 (Not Applicable)
	categorical	Mechanical ventilation given	0 (No); 1 (Yes); 2 (Don't Know); 999 (Not Applicable)
	Date	Date child was first given ventilation	DD/MM/YYYY
	categorical	Continuous Positive Airway Pressure given	0 (No); 1 (Yes); 2 (Don't Know); 999 (Not Applicable)

	categorical	The outcome of the child's hospital stay	0 (Discharged – recovery); 1 (Discharged against medical advice); 2 (Absconded); 3 (Death); 4 (Referred to another facility)

Outcome	categorical	The outcome of the child's illness episode - whether in the hospital or the community setting	0 (Dead); 1 (Alive)
	categorical	If the child died, where did their death take place?	1 (Hospital); 2 (Community)
	categorical	If the child died in the community, was a verbal autopsy carried out?	0 (No); 1 (Yes); 2 (Don't Know)
	categorical	If a verbal autopsy was carried out, was the cause of death listed as pneumonia?	0 (No); 1 (Yes); 2 (Don't Know)
	categorical	Readmission to the hospital within 14 days of discharge.	0 (No); 1 (Yes); 2 (Don't Know)
Clinical Specimen	categorical	Nasal Wash collected	0 (No); 1 (Yes); 2 (Don't Know); 999 (Not Applicable)
	categorical	Nasal Swab collected	0 (No); 1 (Yes); 2 (Don't Know); 999 (Not Applicable)
	categorical	Nasopharyngeal aspirate collected	0 (No); 1 (Yes); 2 (Don't Know); 999 (Not Applicable)
	categorical	Blood culture done	0 (No); 1 (Yes); 2 (Don't Know); 999 (Not Applicable)
	categorical	Pleural aspirate collected	0 (No); 1 (Yes); 2 (Don't Know); 999 (Not Applicable)
Haematology/ Biochemistry	continuous	Haemoglobin count	grams per decilitre (g/dL)
	continuous	Hematocrit level	%
	continuous	White Blood Cell Count	cells/mm^3
	Continuous	Percentage of neutrophils in the blood	%
	continuous	C-Reactive Protein level	mg/Litre
	continuous	Procalcitonin level	mg/Litre
Diagnostic Test	categorical	Result for HIV 1 using ELISA/rapid test (or other antibody tests)	0 (Negative); 1 (Positive); 2 (Don't Know); 999 (Not Performed)
	categorical	Result for HIV 1 using PCR (or other nucleic acid tests)	0 (Negative); 1 (Positive); 2 (Don't Know); 999 (Not Performed)

	categorical	Result for HIV 2 using ELISA/rapid test (or other antibody tests)	0 (Negative); 1 (Positive); 2 (Don't Know); 999 (Not Performed)
	categorical	Result for HIV 2 using PCR (or other nucleic acid tests)	0 (Negative); 1 (Positive); 2 (Don't Know); 999 (Not Performed)
	categorical	Result of a rapid test for Malaria - positive or negative for malaria parasites	0 (Negative); 1 (Positive); 2 (Don't Know); 999 (Not Performed)

	categorical	Result of slide microscopy for Malaria - positive or negative for malaria parasite	0 (Negative); 1 (Positive); 2 (Don't Know); 999 (Not Performed)
	categorical	Result for RSV using ELISA	0 (Negative); 1 (Positive); 2 (Don't Know); 999 (Not Performed)
	categorical	Result for influenza A using ELISA	0 (Negative); 1 (Positive); 2 (Don't Know); 999 (Not Performed)
	categorical	Result for influenza B using ELISA	0 (Negative); 1 (Positive); 2 (Don't Know); 999 (Not Performed)
	categorical	Result for parainfluenza type 1 virus using ELISA	0 (Negative); 1 (Positive); 2 (Don't Know); 999 (Not Performed)
	categorical	Result for parainfluenza type 3 virus using ELISA	0 (Negative); 1 (Positive); 2 (Don't Know); 999 (Not Performed)
	categorical	Result for parainfluenza (other types) virus using ELISA	0 (Negative); 1 (Positive); 2 (Don't Know); 999 (Not Performed)
	categorical	Result for adenovirus using ELISA	0 (Negative); 1 (Positive); 2 (Don't Know); 999 (Not Performed)
	categorical	Result for human metapneumovirus using ELISA	0 (Negative); 1 (Positive); 2 (Don't Know); 999 (Not Performed)
	categorical	Result for rhinovirus (any type) using ELISA	0 (Negative); 1 (Positive); 2 (Don't Know); 999 (Not Performed)
	categorical	Result for RSV using immunofluorescence	0 (Negative); 1 (Positive); 2 (Don't Know); 999 (Not Performed)
	categorical	Result for influenza A using immunofluorescence	0 (Negative); 1 (Positive); 2 (Don't Know); 999 (Not Performed)
	categorical	Result for influenza B using immunofluorescence	0 (Negative); 1 (Positive); 2 (Don't Know); 999 (Not Performed)

	categorical	Result for parainfluenza type 1 virus using immunofluorescence	0 (Negative); 1 (Positive); 2 (Don't Know); 999 (Not Performed)
	categorical	Result for parainfluenza type 3 virus using immunofluorescence	0 (Negative); 1 (Positive); 2 (Don't Know); 999 (Not Performed)
	categorical	Result for parainfluenza (other types) virus using immunofluorescence	0 (Negative); 1 (Positive); 2 (Don't Know); 999 (Not Performed)
	categorical	Result for adenovirus using immunofluorescence	0 (Negative); 1 (Positive); 2 (Don't Know); 999 (Not Performed)
	categorical	Result for human metapneumovirus using immunofluorescence	0 (Negative); 1 (Positive); 2 (Don't Know); 999 (Not Performed)
	categorical	Result for rhinovirus (any type) using immunofluorescence	0 (Negative); 1 (Positive); 2 (Don't Know); 999 (Not Performed)
	categorical	Result for RSV using PCR	0 (Negative); 1 (Positive); 2 (Don't Know); 999 (Not Performed)
	categorical	Result for influenza A using PCR	0 (Negative); 1 (Positive); 2 (Don't Know); 999 (Not Performed)
	categorical	Result for influenza B using PCR	0 (Negative); 1 (Positive); 2 (Don't Know); 999 (Not Performed)
	categorical	Result for parainfluenza type 1 virus using PCR	0 (Negative); 1 (Positive); 2 (Don't Know); 999 (Not Performed)
	categorical	Result for parainfluenza type 3 virus using PCR	0 (Negative); 1 (Positive); 2 (Don't Know); 999 (Not Performed)
	categorical	Result for parainfluenza (other types) virus using PCR	0 (Negative); 1 (Positive); 2 (Don't Know); 999 (Not Performed)
	categorical	Result for adenovirus using PCR	0 (Negative); 1 (Positive); 2 (Don't Know); 999 (Not Performed)
	categorical	Result for human metapneumovirus using PCR	0 (Negative); 1 (Positive); 2 (Don't Know); 999 (Not Performed)
	categorical	Result for rhinovirus (any type) using PCR	0 (Negative); 1 (Positive); 2 (Don't Know); 999 (Not Performed)
	categorical	Result for *Streptococcus pneumoniae* using blood culture	0 (Negative); 1 (Positive); 2 (Don't Know); 999 (Not Performed)

	categorical	Result for *Haemophilus influenzae* type b using blood culture	0 (Negative); 1 (Positive); 2 (Don't Know); 999 (Not Performed)
	categorical	Result for *Staphylococcus aureus* using blood culture	0 (Negative); 1 (Positive); 2 (Don't Know); 999 (Not Performed)
	categorical	Result for *Klebsiella pneumoniae* using blood culture	0 (Negative); 1 (Positive); 2 (Don't Know); 999 (Not Performed)
	categorical	Result for *Streptococcus pneumoniae* using pleural aspirate	0 (Negative); 1 (Positive); 2 (Don't Know); 999 (Not Performed)
	categorical	Result for *Haemophilus influenzae* type b using pleural aspirate	0 (Negative); 1 (Positive); 2 (Don't Know); 999 (Not Performed)
	categorical	Result for *Staphylococcus aureus* using pleural aspirate	0 (Negative); 1 (Positive); 2 (Don't Know); 999 (Not Performed)
	categorical	Result for *Klebsiella pneumoniae* using pleural aspirate	0 (Negative); 1 (Positive); 2 (Don't Know); 999 (Not Performed)
In-Hospital Followup	continuous	Respiratory rate 1 day (24 hours) after hospitalisation, then D2D8	Breaths/Minute
	continuous	Temperature 1 day (24 hours) after hospitalisation, then D2-D8	Degrees Celsius
	categorical	Chest indrawing/Subcostal retractions 1 day (24 hours) after hospitalisation, then D2-D8	0 (No); 1 (Yes); 2 (Don't Know)
	categorical	Inability to drink 1 day (24 hours) after hospitalisation, then D2-D8	0 (No); 1 (Yes); 2 (Don't Know)
	categorical	Difficulty feeding 1 day (24 hours) after hospitalisation, then D2-D8	0 (No); 1 (Yes); 2 (Don't Know)
	categorical	Convulsions/seizures 1 day (24 hours) after hospitalisation, then D2-D8	0 (No); 1 (Yes); 2 (Don't Know)
	categorical	Cyanosis 1 day (24 hours) after hospitalisation, then D2-D8	0 (No); 1 (Yes); 2 (Don't Know)
	categorical	Head nodding/bobbing 1 day (24 hours) after hospitalisation, then D2-D8	0 (No); 1 (Yes); 2 (Don't Know)
	categorical	Decreased consciousness 1 day (24 hours) after hospitalisation. Includes dull/unresponsive, difficult to wake, lethargy, prostration, then D2-D8	0 (No); 1 (Yes); 2 (Don't Know)
	categorical	Irritable/agitated 1 day (24 hours) after hospitalisation, then D2-D8	0 (No); 1 (Yes); 2 (Don't Know)

	continuous	Oxygen saturation 1 day (24 hours) after hospitalisation, then D2-D8	%
	Categorical	Was oxygen saturation measured on room air or supplementary oxygen, then D2-D8	1 (Room Air); 2 (Supplementary Oxygen); 3 (Don't Know)
	continuous	Heart rate 1 day (24 hours) after hospitalisation, then D2-D8	Beats/Minute

Table S3. Variable Dictionary: Data Collection for Hospital-based Studies

	Type	Explanation	Format of return
Demographic Information	descriptive	Admission ID for the child - please enter one hospital admission per row.	Free text - please enter this however you recorded it.
	descriptive	Please enter an ID code for the child	Free text - please enter this however you recorded it.
	Descriptive	Location of study site where the child was assessed.	Free text – Please enter the Location of the study site.
	date	Date of birth of the child	DD/MM/YYYY
	continuous	Age of child	Days
	categorical	Sex of child	0 (Male); 1 (Female); 2 (Don't Know)
	categorical	Maternal HIV status	0 (Negative); 1 (Positive); 2 (Don't Know)
General Medical History	categorical	Any previous hospitalisations in the past 2 weeks?	0 (No); 1 (Yes); 2 (Don't Know); 999 (Not Applicable)
	Categorical	Was this previous hospitalisation for pneumonia?	0 (No); 1 (Yes); 2 (Don't Know); 999 (Not Applicable)
History of Current Illness	date	Date of onset of current illness	DD/MM/YYYY
	categorical	History of Cough within the past 14 days	0 (No); 1 (Yes); 2 (Don't Know); 999 (Not Applicable)
	continuous	Duration of cough	Days
	categorical	History of difficulty breathing within the past 14 days	0 (No); 1 (Yes); 2 (Don't Know); 999 (Not Applicable)

	continuous	Duration of difficulty breathing	Days
	categorical	History of fever (as reported by caregiver) within the past 14 days	0 (No); 1 (Yes); 2 (Don't Know); 999 (Not Applicable)
	continuous	Duration of fever (as reported by caregiver)	Days
	categorical	History of inability to drink or breastfeed within the past 14 days	0 (No); 1 (Yes); 2 (Don't Know); 999 (Not Applicable)
	continuous	Duration of inability to drink or breastfeed	Days
	categorical	History of convulsions/seizures within the past 14 days	0 (No); 1 (Yes); 2 (Don't Know); 999 (Not Applicable)
	continuous	Duration of convulsions/seizure	Days
	categorical	History of vomiting everything within the past 14 days	0 (No); 1 (Yes); 2 (Don't Know); 999 (Not Applicable)
	continuous	Duration of vomiting everything	Days
	categorical	History of decreased level of consciousness within past 14 days. This includes a history of being dull/unresponsive, abnormally sleepy, difficult to wake, lethargy and prostration.	0 (No); 1 (Yes); 2 (Don't Know); 999 (Not Applicable)
	continuous	Duration of decreased level of consciousness	Days
	categorical	Use of antibiotics within the past 14 days	0 (No); 1 (Yes); 2 (Don't Know); 999 (Not Applicable)
Examinations before Hospital Admission	categorical	Were any examinations done on the child before hospitalisation? E.g. at a clinic.	0 (No); 1 (Yes); 2 (Don't Know)
	date	Date of examination before hospitalisation	DD/MM/YYYY
	continuous	Respiratory rate (measured when child calm) at the examination before hospitalisation	Breaths/Minute
	continuous	The temperature at the examination before hospitalisation	Degrees Celsius
	categorical	The site at which temperature was measured	1 (oral); 2 (axillary); 3 (rectal); 4 (tympanic); 5 (Don't know)
	categorical	Chest indrawing at the examination before hospitalisation	0 (No); 1 (Yes); 2 (Don't Know); 999 (Not Applicable)
	categorical	Inability to drink at the examination before hospitalisation	0 (No); 1 (Yes); 2 (Don't Know); 999 (Not Applicable)

	categorical	Difficulty breastfeeding at the examination before hospitalisation	0 (No); 1 (Yes); 2 (Don't Know); 999 (Not Applicable)
	categorical	Convulsions/seizures at the examination before hospitalisation	0 (No); 1 (Yes); 2 (Don't Know); 999 (Not Applicable)
	categorical	Cyanosis at the examination before hospitalisation	0 (No); 1 (Yes); 2 (Don't Know); 999 (Not Applicable)
	categorical	Head nodding/bobbing at the examination before hospitalisation	0 (No); 1 (Yes); 2 (Don't Know); 999 (Not Applicable)
	categorical	Decreased consciousness at the examination before hospitalisation. Includes dull/unresponsive, abnormally sleepy, difficult to wake, lethargy and prostration.	0 (No); 1 (Yes); 2 (Don't Know); 999 (Not Applicable)
	categorical	Irritable/agitated at the examination before hospitalisation	0 (No); 1 (Yes); 2 (Don't Know); 999 (Not Applicable)
	continuous	Oxygen saturation at the examination before hospitalisation	%

	continuous	Heart rate at the examination before hospitalisation	Beats/Minute
Examination on Hospital Admission	categorical	Child hospitalized	0 (No); 1 (Yes); 2 (Don't Know); 999 (Not Applicable)
	date	Date of hospital admission	DD/MM/YYYY
Vital Signs at Hospital	continuous	Respiratory Rate (measurement 1) on admission to the hospital (measured when child calm)	Breaths/Minute
	continuous	Respiratory Rate (measurement 2, if taken) on admission to the hospital (measured when child calm)	Breaths/Minute
	continuous	The temperature on admission to the hospital	Degrees Celsius
	categorical	The site at which temperature was measured	1 (oral); 2 (axillary); 3 (rectal); 4 (tympanic); 5 (Don't know); 999 (Not Applicable)
	continuous	Heart Rate on admission to the hospital	Beats/Minute
Respiratory Signs at Hospital	categorical	Cough on admission to the hospital	0 (No); 1 (Yes); 2 (Don't Know); 999 (Not Applicable)
	categorical	Difficulty breathing on admission to the hospital	0 (No); 1 (Yes); 2 (Don't Know); 999 (Not Applicable)

	categorical	Apnoea on admission to the hospital	0 (No); 1 (Yes); 2 (Don't Know); 999 (Not Applicable)
	categorical	Audible wheeze on admission to the hospital	0 (No); 1 (Yes); 2 (Don't Know); 999 (Not Applicable)
	categorical	Audible stridor on admission to the hospital	0 (No); 1 (Yes); 2 (Don't Know); 999 (Not Applicable)
	categorical	Audible grunting on admission to the hospital	0 (No); 1 (Yes); 2 (Don't Know); 999 (Not Applicable)
	categorical	Nasal flaring on admission to the hospital	0 (No); 1 (Yes); 2 (Don't Know); 999 (Not Applicable)
	categorical	Presence of lower chest indrawing/subcostal retractions on admission to the hospital	0 (No); 1 (Yes); 2 (Don't Know); 999 (Not Applicable)
	categorical	Presence of intercostal indrawing/intercostal retractions on admission to the hospital	0 (No); 1 (Yes); 2 (Don't Know); 999 (Not Applicable)
	categorical	The runny nose on admission to the hospital	0 (No); 1 (Yes); 2 (Don't Know); 999 (Not Applicable)
	Categorical	Ear discharge on admission to the hospital	0 (No); 1 (Yes); 2 (Don't Know); 999 (Not Applicable)

Auscultatory Signs at hospital admission	categorical	Bronchial breathing on auscultation at the hospital admission	0 (No); 1 (Yes); 2 (Don't Know); 999 (Not Applicable)
	categorical	Crepitations/rales/crackles present on auscultation at the hospital admission	0 (No); 1 (Yes); 2 (Don't Know); 999 (Not Applicable)
	categorical	Wheeze/rhonchi on auscultation at the hospital admission	0 (No); 1 (Yes); 2 (Don't Know); 999 (Not Applicable)
Danger Signs at hospital admission	categorical	Inability to drink or feed (including breastfeeding) on admission to the hospital	0 (No); 1 (Yes); 2 (Don't Know); 999 (Not Applicable)
	categorical	Convulsions/seizures on admission to the hospital	0 (No); 1 (Yes); 2 (Don't Know); 999 (Not Applicable)
	categorical	Cyanosis on admission to the hospital	0 (No); 1 (Yes); 2 (Don't Know); 999 (Not Applicable)
	categorical	Head nodding/bobbing on admission to the hospital	0 (No); 1 (Yes); 2 (Don't Know); 999 (Not Applicable)

	categorical	Decreased consciousness on admission to the hospital. Includes dull/unresponsive, abnormally sleepy, difficult to wake, lethargy and prostration.	0 (No); 1 (Yes); 2 (Don't Know); 999 (Not Applicable)
	categorical	The decreased activity level upon admission to the hospital	0 (No); 1 (Yes); 2 (Don't Know); 999 (Not Applicable)
	categorical	Irritable/agitated on admission to the hospital	0 (No); 1 (Yes); 2 (Don't Know); 999 (Not Applicable)
General Examination at Hospital	continuous	Weight on admission to the hospital	kg
	continuous	Height on admission to the hospital	cm
	continuous	Mid Upper Arm Circumference on admission to the hospital	cm
	continuous	Oxygen saturation on admission to the hospital	%
	Categorical	Was oxygen saturation measured on room air or supplementary oxygen	1 (Room Air); 2 (supplementary oxygen); 3 (Don’t Know); 999 (Not Applicable)
Chest X-ray Findings	categorical	Chest X-ray taken	0 (No); 1 (Yes); 2 (Don't Know)
	date	The date on which child's chest x-ray was taken	DD/MM/YYYY
	categorical	Chest X-ray quality	0 (Uninterpretable); 1 (Suboptimal); 2 (Adequate)
	categorical	Number of readers interpreting the chest x-ray	1 (1 reader); 2 (2 readers); 3 (3 readers); 4 (2 readers and 1 arbiter)
	categorical	Chest x-ray findings meeting Primary-Endpoint Criteria (see Definitions document)	0 (No); 1 (Yes); 2 (Don't Know)
	categorical	Chest x-ray findings showing lobar pneumonia/consolidation	0 (No); 1 (Yes); 2 (Don't Know)
	categorical	Pleural effusion present on chest x-ray	0 (No); 1 (Yes); 2 (Don't Know)
	categorical	Abscess present on chest x-ray	0 (No); 1 (Yes); 2 (Don't Know)
	categorical	Other abnormal findings were present on the chest x-ray. e.g., atelectasis/pneumatocele/pneumothorax	0 (No); 1 (Yes); 2 (Don't Know)
	categorical	Any other infiltrate present on chest x-ray	0 (No); 1 (Yes); 2 (Don't Know)
	categorical	Chest X-ray normal	0 (No); 1 (Yes); 2 (Don't Know)

Vaccination Status	categorical	Number of doses of pentavalent[2] vaccination child has received	0 (0 doses); 1 (1 dose); 2 (2 doses); 3 (3 doses); 4 (doses not known; vaccination up to date, as verified by vaccination card); 999 (Not Applicable)
	categorical	Number of doses of PCV vaccination child has received	0 (0 doses); 1 (1 dose); 2 (2 doses); 3 (3 doses); 4 (doses not known; vaccination up to date, as verified by vaccination card); 999 (Not Applicable)
	categorical	Number of doses of Hib vaccination child has received	0 (0 doses); 1 (1 dose); 2 (2 doses); 3 (3 doses); 4 (doses not known; vaccination up to date, as verified by vaccination card); 999 (Not Applicable)
	categorical	Number of doses of DPT (Diphtheria, Pertussis, Tetanus) vaccination child has received	0 (0 doses); 1 (1 dose); 2 (2 doses); 3 (3 doses); 4 (doses not known; vaccination up to date, as verified by vaccination card); 999 (Not Applicable)
	categorical	Number of doses of Measles vaccination child has received	0 (0 doses); 1 (1 dose); 2 (2 doses); 3 (doses not known; vaccination up to date, as verified by vaccination card); 999 (Not Applicable)

Treatment	Categorical	Diagnosis at initial examination at the hospital. Please specify.	1 (Pneumonia - all severities); 2 (Bronchiolitis); 3 (Bronchitis); 4 (Wheezy Bronchitis); 5 (Asthma); 6 (Other - specify below)
	descriptive	If 'other' is listed as the initial examination diagnosis in DIAG_ADM, please specify the diagnosis here.	Free text

[2] The pentavalent vaccine includes DPT (Diphtheria, Pertussis, Tetanus), Hepatitis B, and Hib vaccines.

	Categorical	Diagnosis at discharge, or final diagnosis. Please specify.	1 (Pneumonia - all severities); 2 (Bronchiolitis); 3 (Bronchitis); 4 (Wheezy Bronchitis); 5 (Asthma); 6 (Other - specify below)
	descriptive	If 'other' is listed as discharge diagnosis in DIAG_DIS, please specify the diagnosis here.	Free text
	continuous	Duration of hospital stay	Days
	categorical	Bronchodilator challenge received (see Definitions document)	0 (No); 1 (Yes); 2 (Don't Know); 999 (Not Applicable)
	categorical	Result of bronchodilator challenge - fail or success	0 (Fail); 1 (Success); 2 (Don't Know); 999 (Not Applicable)
	categorical	Antibiotics are given to the child	0 (No); 1 (Yes); 2 (Don't Know)
	Continuous	Duration of antibiotic treatment	Days
	categorical	Was there a change of antibiotic treatment given to the child(s)	0 (No); 1 (Yes); 2 (Don't Know); 999 (Not Applicable)
	date	Date of change of antibiotic(s)	DD/MM/YYYY
	categorical	Reason for change of antibiotic(s)	0 (persistence of signs); 1 (clinical deterioration - development of new danger signs); 2 (Clinical deterioration - fall in oxygen saturation); 3 (Failure to improve); 4 (Other - please specify below); 5 (Don't Know); 999 (Not Applicable)
	descriptive	If 'other' is listed as the reason for the change in ABX_CHREAS, please specify the reason here	Free text
	categorical	Was the course of antibiotics completed successfully?	0 (No); 1 (Yes); 2 (Don't Know); 999 (Not Applicable)
	categorical	Use of bronchodilator during illness	0 (No); 1 (Yes); 2 (Don't Know); 999 (Not Applicable)
	categorical	Supplemental oxygen was given	0 (No); 1 (Yes); 2 (Don't Know); 999 (Not Applicable)
	date	Date child is first given supplemental oxygen	DD/MM/YYYY

	continuous	Duration of supplemental oxygen use	Hours
	categorical	ICU/intensive admission or care	0 (No); 1 (Yes); 2 (Don't Know); 999 (Not Applicable)
	categorical	Need for ventilation (any - manual or mechanical), regardless of availability. Includes children who needed ventilation but were not given it.	0 (No); 1 (Yes); 2 (Don't Know); 999 (Not Applicable)
	categorical	Manual ventilation was given	0 (No); 1 (Yes); 2 (Don't Know); 999 (Not Applicable)
	categorical	Mechanical ventilation was given	0 (No); 1 (Yes); 2 (Don't Know); 999 (Not Applicable)
	Date	Date child was first given ventilation	DD/MM/YYYY
	categorical	Continuous Positive Airway Pressure was given	0 (No); 1 (Yes); 2 (Don't Know); 999 (Not Applicable)
Outcome	categorical	Child's outcome	0 (Discharged - recovery); 1 (Discharged against medical advice); 2 (Absconded); 3 (Death); 4 (Referred to another facility)
	categorical	Readmission to the hospital within 14 days of discharge.	0 (No); 1 (Yes); 2 (Don't Know)
Clinical Specimen	categorical	Nasal Wash collected	0 (No); 1 (Yes); 2 (Don't Know); 999 (Not Applicable)
	categorical	Nasal Swab collected	0 (No); 1 (Yes); 2 (Don't Know); 999 (Not Applicable)
	categorical	Nasopharyngeal aspirate collected	0 (No); 1 (Yes); 2 (Don't Know); 999 (Not Applicable)
	categorical	Blood culture done	0 (No); 1 (Yes); 2 (Don't Know); 999 (Not Applicable)
	categorical	Pleural aspirate collected	0 (No); 1 (Yes); 2 (Don't Know); 999 (Not Applicable)
Haematology/ Biochemistry	continuous	Haemoglobin count	grams per decilitre (g/dL)
	continuous	Hematocrit level	%
	continuous	White Blood Cell Count	cells/mm^3

	Continuous	Percentage of neutrophils in the blood	%
	continuous	C-Reactive Protein level	mg/Litre
	continuous	Procalcitonin level	mg/Litre
Diagnostic Test	categorical	Result for HIV 1 using ELISA/rapid test (or other antibody tests)	0 (Negative); 1 (Positive); 2 (Don't Know); 999 (Not Performed)
	categorical	Result for HIV 1 using PCR (or other nucleic acid tests)	0 (Negative); 1 (Positive); 2 (Don't Know); 999 (Not Performed)
	categorical	Result for HIV 2 using ELISA/rapid test (or other antibody tests)	0 (Negative); 1 (Positive); 2 (Don't Know); 999 (Not Performed)
	categorical	Result for HIV 2 using PCR (or other nucleic acid tests)	0 (Negative); 1 (Positive); 2 (Don't Know); 999 (Not Performed)
	categorical	Result of a rapid test for Malaria - positive or negative for malaria parasites	0 (Negative); 1 (Positive); 2 (Don't Know); 999 (Not Performed)
	categorical	Result of slide microscopy for Malaria - positive or negative for malaria parasite	0 (Negative); 1 (Positive); 2 (Don't Know); 999 (Not Performed)
	categorical	Result for RSV using ELISA	0 (Negative); 1 (Positive); 2 (Don't Know); 999 (Not Performed)
	categorical	Result for influenza A using ELISA	0 (Negative); 1 (Positive); 2 (Don't Know); 999 (Not Performed)
	categorical	Result for influenza B using ELISA	0 (Negative); 1 (Positive); 2 (Don't Know); 999 (Not Performed)
	categorical	Result for parainfluenza type 1 virus using ELISA	0 (Negative); 1 (Positive); 2 (Don't Know); 999 (Not Performed)
	categorical	Result for parainfluenza type 3 virus using ELISA	0 (Negative); 1 (Positive); 2 (Don't Know); 999 (Not Performed)
	categorical	Result for parainfluenza (other types) virus using ELISA	0 (Negative); 1 (Positive); 2 (Don't Know); 999 (Not Performed)
	categorical	Result for adenovirus using ELISA	0 (Negative); 1 (Positive); 2 (Don't Know); 999 (Not Performed)
	categorical	Result for human metapneumovirus using ELISA	0 (Negative); 1 (Positive); 2 (Don't Know); 999 (Not Performed)
	categorical	Result for rhinovirus (any type) using ELISA	0 (Negative); 1 (Positive); 2 (Don't Know); 999 (Not Performed)

	categorical	Result for RSV using immunofluorescence	0 (Negative); 1 (Positive); 2 (Don't Know); 999 (Not Performed)

	categorical	Result for influenza A using immunofluorescence	0 (Negative); 1 (Positive); 2 (Don't Know); 999 (Not Performed)
	categorical	Result for influenza B using immunofluorescence	0 (Negative); 1 (Positive); 2 (Don't Know); 999 (Not Performed)
	categorical	Result for parainfluenza type 1 virus using immunofluorescence	0 (Negative); 1 (Positive); 2 (Don't Know); 999 (Not Performed)
	categorical	Result for parainfluenza type 3 virus using immunofluorescence	0 (Negative); 1 (Positive); 2 (Don't Know); 999 (Not Performed)
	categorical	Result for parainfluenza (other types) virus using immunofluorescence	0 (Negative); 1 (Positive); 2 (Don't Know); 999 (Not Performed)
	categorical	Result for adenovirus using immunofluorescence	0 (Negative); 1 (Positive); 2 (Don't Know); 999 (Not Performed)
	categorical	Result for human metapneumovirus using immunofluorescence	0 (Negative); 1 (Positive); 2 (Don't Know); 999 (Not Performed)
	categorical	Result for rhinovirus (any type) using immunofluorescence	0 (Negative); 1 (Positive); 2 (Don't Know); 999 (Not Performed)
	categorical	Result for RSV using PCR	0 (Negative); 1 (Positive); 2 (Don't Know); 999 (Not Performed)
	categorical	Result for influenza A using PCR	0 (Negative); 1 (Positive); 2 (Don't Know); 999 (Not Performed)
	categorical	Result for influenza B using PCR	0 (Negative); 1 (Positive); 2 (Don't Know); 999 (Not Performed)
	categorical	Result for parainfluenza type 1 virus using PCR	0 (Negative); 1 (Positive); 2 (Don't Know); 999 (Not Performed)
	categorical	Result for parainfluenza type 3 virus using PCR	0 (Negative); 1 (Positive); 2 (Don't Know); 999 (Not Performed)
	categorical	Result for parainfluenza (other types) virus using PCR	0 (Negative); 1 (Positive); 2 (Don't Know); 999 (Not Performed)
	categorical	Result for adenovirus using PCR	0 (Negative); 1 (Positive); 2 (Don't Know); 999 (Not Performed)

	categorical	Result for human metapneumovirus using PCR	0 (Negative); 1 (Positive); 2 (Don't Know); 999 (Not Performed)
	categorical	Result for rhinovirus (any type) using PCR	0 (Negative); 1 (Positive); 2 (Don't Know); 999 (Not Performed)
	categorical	Result for *Streptococcus pneumoniae* using blood culture	0 (Negative); 1 (Positive); 2 (Don't Know); 999 (Not Performed)
	categorical	Result for *Haemophilus influenzae* type b using blood culture	0 (Negative); 1 (Positive); 2 (Don't Know); 999 (Not Performed)
	categorical	Result for *Staphylococcus aureus* using blood culture	0 (Negative); 1 (Positive); 2 (Don't Know); 999 (Not Performed)
	categorical	Result for *Klebsiella pneumoniae* using blood culture	0 (Negative); 1 (Positive); 2 (Don't Know); 999 (Not Performed)
	categorical	Result for *Streptococcus pneumoniae* using pleural aspirate	0 (Negative); 1 (Positive); 2 (Don't Know); 999 (Not Performed)
	categorical	Result for *Haemophilus influenzae* type b using pleural aspirate	0 (Negative); 1 (Positive); 2 (Don't Know); 999 (Not Performed)
	categorical	Result for *Staphylococcus aureus* using pleural aspirate	0 (Negative); 1 (Positive); 2 (Don't Know); 999 (Not Performed)
	categorical	Result for *Klebsiella pneumoniae* using pleural aspirate	0 (Negative); 1 (Positive); 2 (Don't Know); 999 (Not Performed)
In-Hospital Followup D1 to D14	continuous	Respiratory rate 1 day (24 hours) after hospitalisation	Breaths/Minute
	continuous	Temperature 1 day (24 hours) after hospitalisation	Degrees Celsius
	categorical	Chest indrawing/Subcostal retractions 1 day (24 hours) after hospitalisation	0 (No); 1 (Yes); 2 (Don't Know)
	categorical	Inability to drink 1 day (24 hours) after hospitalisation	0 (No); 1 (Yes); 2 (Don't Know)
	categorical	Difficulty feeding 1 day (24 hours) after hospitalisation	0 (No); 1 (Yes); 2 (Don't Know)
	categorical	Convulsions/seizures 1 day (24 hours) after hospitalisation	0 (No); 1 (Yes); 2 (Don't Know)
	categorical	Cyanosis 1 day (24 hours) after hospitalisation	0 (No); 1 (Yes); 2 (Don't Know)
	categorical	Head nodding/bobbing 1 day (24 hours) after hospitalisation	0 (No); 1 (Yes); 2 (Don't Know)
	categorical	Decreased consciousness 1 day (24 hours) after hospitalisation. Includes dull/unresponsive, difficult to wake, lethargy, and prostration.	0 (No); 1 (Yes); 2 (Don't Know)

	categorical	Irritable/agitated 1 day (24 hours) after hospitalisation	0 (No); 1 (Yes); 2 (Don't Know)
	continuous	Oxygen saturation 1 day (24 hours) after hospitalisation	%
	Categorical	Was oxygen saturation measured on room air or supplementary oxygen?	1 (Room Air); 2 (Supplementary Oxygen); 3 (Don't Know)
	continuous	Heart rate 1 day (24 hours) after hospitalisation	Beats/Minute

Table S4. Study Description Template

**Study Description Data Items**
Lead Investigator / Main contact
Study Location
Age range
Case definition for pneumonia
Inclusion criteria
Exclusion criteria
Study setting (community/hospital/community with hospital referral)
Sample size
Radiology data available (Y/N)
Eligibility criteria for chest Xray in the child hospitalised for ALRI
Case definition used for primary endpoint pneumonia
Number of readers for chest radiograph
Was the standard WHO training provided to all readers and the arbitrer (Y/N)
Pulse oximetry data available (Y/N)
Make of pulse oximeter used and type of probe used
Number of respiratory rate readings taken at time of admission
The interval at which RR readings were taken
Frequency of follow-up (e.g. 12 h/24 h/36 h/48 h)
Follow-up data available
Number of pneumonia deaths
The altitude of the study site
Standard first-line antibiotic treatment given to children (when necessary)

Standard second-line antibiotic treatment given to children (when necessary)
Hib/ PCV Immunisation coverage
Prevalence of malaria (*Plasmodium falciparum*)
Paediatric HIV prevalence
Infant mortality rate
Under-five mortality rate
If community-based study, verbal autopsy data is available (Y/N/NA)
